# Maternal Diet and Weight at 3 Months Postpartum Following a Pregnancy Intervention with a Low Glycaemic Index Diet: Results from the ROLO Randomised Control Trial

**DOI:** 10.3390/nu6072946

**Published:** 2014-07-23

**Authors:** Mary K. Horan, Ciara A. McGowan, Eileen R. Gibney, Jean M. Donnelly, Fionnuala M. McAuliffe

**Affiliations:** 1 UCD Obstetrics and Gynaecology, School of Medicine and Medical Science, University College Dublin, National Maternity Hospital, Dublin 2, Ireland; E-Mails: mary.horan@ucdconnect.ie (M.K.H.); cmcgowa@gmail.com (C.A.M.); jeandonnelly80@yahoo.co.uk (J.M.D.); 2 UCD Institute of Food and Health, University College Dublin, Dublin 4, Ireland; E-Mail: eileen.gibney@ucd.ie

**Keywords:** low glycaemic index, postpartum, maternal diet, pregnancy, weight, post-intervention, ROLO study

## Abstract

Pregnancy increases the risk of being overweight at a later time period, particularly when there is excessive gestational weight gain. There remains a paucity of data into the effect of low glycaemic index (GI) pregnancy interventions postpartum. Aim: To examine the impact of a low glycaemic index diet during pregnancy on maternal diet 3 months postpartum. Methodology: This analysis examined the diet, weight and lifestyle of 460 participants of the ROLO study 3 months postpartum. Questionnaires on weight, physical activity, breastfeeding, supplement use, food label reading and dietary habits were completed. Results: The intervention group had significantly greater weight loss from pre-pregnancy to 3 months postpartum than the control group (1.3 *vs.* 0.1 kg, *p* = 0.022). The intervention group reported greater numbers following a low glycaemic index diet (*p* < 0.001) and reading food labels (*p* = 0.032) and had a lower glycaemic load (GL) (128 *vs.* 145, *p* = 0.014) but not GI (55 *vs.* 55, *p* = 0.809) than controls. Conclusions: Low GI dietary interventions in pregnancy result in improved health-behaviours and continued reported compliance at 3 months postpartum possibly through lower dietary GL as a result of portion control. Greater levels of weight loss from pre-pregnancy to 3 months postpartum in the intervention group may have important positive implications for overweight and obesity.

## 1. Introduction

Low glycaemic index (GI) diet in pregnancy has been found by some studies to reduce hyperglycaemia and risk of macrosomia [1,2,3]. The ROLO (Randomised cOntrol trial of LOw glycaemic index diet *versus* no dietary intervention to prevent recurrence of fetal macrosomia) study was the first large randomised control trial to examine the effect of low GI diet in pregnancy on maternal and infant outcomes [4]. One third of women whose first infant was macrosomic have been found to give birth to another macrosomic infant in their second pregnancy [5]. There is evidence to suggest that maternal hyperglycaemia even below limits for diagnosis of gestational diabetes may increase risk of macrosomia [6]. Therefore, the ROLO study examined secondogravid women who had previously given birth to a macrosomic (>4 kg) infant. 800 women were randomised to receive either low glycaemic index dietary advice or usual antenatal care which did not include dietary advice in an attempt to reduce postprandial peaks in blood glucose [4]. The dietary intervention involved a single 1–2 h session with a fully trained dietician for groups of 2–6 women. Verbal and written information and advice was given on overall healthy eating and low GI diet. No advice was given regarding appropriate gestational weight gain or energy intake although women were advised that there was no need to “eat for two”. Although women were given only one dietary advice session, they were able to contact the dietician subsequently with any questions or difficulties. The primary outcome was reduction in rates of macrosomia while the secondary outcomes were a reduction in excess gestational weight gain and attenuation of the normal pregnancy-related increase glucose intolerance [4]. Findings from the ROLO study showed that low GI intervention resulted in reduced gestational weight gain and improved compliance with the IOM guidelines for gestational weight gain [7], reduced glycaemic index and load, and improved glycaemic control following challenge with a 50 g glucose load and diet quality, *i.e.*, increased wholegrain, fibre, and micronutrient intake and reduced overall energy, high-energy beverage and refined cereal intake. No effect of the low GI diet was noted on birthweight or macrosomia [8,9]. 

GI is used to classify different carbohydrate foods according to glycaemic response GI is defined as “the incremental area under the blood glucose response curve of a test food containing 50 g available carbohydrate expressed as a percentage of the response to the same amount of available carbohydrate from a reference food”, the reference food usually being glucose or white bread [10]. GL was developed to take into account the actual portions of carbohydrate foods eaten. GL is the product of the GI and its actual carbohydrate content (expressed as grams carbohydrate per 100 grams overall food) [11]. Some studies have examined the effect of low GI “healthy-eating” pregnancy interventions postpartum in women with GDM with poor adherence to lifestyle change observed [12,13]. Fehler *et al.* [12] found that dietary changes made during GDM pregnancy were not sustained at 6 months postpartum and Stage *et al.* [13] found that while the women reported being concerned about developing type II diabetes, more women had gained weight than had lost weight at 11–42 months postpartum and physical activity levels had not improved since pregnancy. However, Stage *et al.* [13] also found that the number of women reporting consuming a high fat diet had decreased from that reported pre-pregnancy. Another recent study by Louie *et al.* [14] of low GI diet *vs.* traditional high-fibre dietary advice in GDM pregnancy also found no difference in postpartum weight retention at 3 months [14]. Besides these studies, there remains little data in this area, particularly in non-GDM pregnancies although an observational study by Knudsen *et al.* [15] found that high dietary GL in pregnancy was associated with increased postpartum weight retention at 18 months in overweight and obese women. Pregnancy has been found to be a risk factor for increased overweight postpartum [16] largely mediated through excess gestational weight gain [17]. Therefore, pregnancy interventions that improve diet at a time when women are very motivated to improve their health for the benefit of the fetus may be helpful in effecting longer term dietary changes and health benefits [18]. The aim of this study was to examine the effect of the ROLO study low GI intervention at 3 months postpartum. 

## 2. Experimental Section

This research adhered to the principles of the Declaration of Helsinki and received institutional ethical approval. Data from the ROLO study (*n* = 460) was used in this analysis. Lifestyle and dietary habit questionnaires were completed at 3 months postpartum including questions on physical activity, breastfeeding, supplement use, food label reading and dietary change since the intervention began. Self-reported maternal and infant weights were also recorded. Physical activity was recorded as number of 20 min intervals of physical activity per week. An explanation of the intensity of different levels of physical activity and examples of each were given to participants when completing physical activity questions as follows; strenuous physical activity was defined as physical activity that resulted in “rapid heartbeat”, e.g., running, jogging, hurling, camogie, football, soccer, squash, basketball, judo, roller skating, vigorous swimming, vigorous long distance cycling, advanced aerobics; moderate physical activity was defined as “not exhausting”, e.g., fast walking, tennis, badminton, easy swimming, easy cycling, popular and folk dancing, intermediate aerobics, heavy gardening; and mild physical activity was defined as “minimal effort” physical activity, e.g., yoga, golf, easy walking, fishing from river bank, bowling, beginners aerobics, archery, light gardening. Participants were asked yes/no questions regarding whether they had made dietary changes since participating in the ROLO study, whether they were following a weight-reducing diet and whether they took dietary supplements. Participants were also asked whether they read food labels always, sometimes or never and if they answered that they always or sometimes read food labels this was coded as an affirmative answer and they were then asked what information they looked for on these food labels, *i.e.*, ingredients, nutrient-content, calorie-content, food weight, additive-content and/or serving size.

### Food Frequency Questionnaire

Dietary intake over the past 3 months was examined using a condensed version of the self-administered 170 item SLAN (Survey of Lifestyle, Attitudes and Nutrition in Ireland) food frequency questionnaire (FFQ) [9]. The FFQ was condensed to include only questions on foods which affect dietary GI, *i.e.*, moderate- to high-carbohydrate content foods, and was therefore used only to compare the GI and GL values of the control and intervention groups at 3 months postpartum. The condensed FFQ contained 88 food and beverage items which had a carbohydrate content >3 g per 100 g. The frequency of consumption was coded into nine categories ranging from consumed “never or less than once per month” to consumed “six or more times per day”. This condensed FFQ was validated against the full SLAN FFQ for GI and therefore it was also possible to compare postpartum and early-pregnancy GI. Validation was carried out using a subgroup of 46 participants, half of whom were asked to first complete the original unmodified SLAN FFQ and then to complete the condensed FFQ and half of whom completed the FFQs in the reverse order. Reported frequency of consumption of the 88 food and beverage items and of the GI and GL values were then compared between the unmodified and condensed FFQs and also between participants who had completed the condensed FFQ before the unmodified FFQ and those that had completed the FFQs in the reverse order.

The coded frequencies of consumption (1–9) were entered into QBuilder V2.0 (Tinuviel Software, Anglesey, UK) and this software was used to analyse the food frequency questionnaire data. Since QBuilder contains the GI values of foods based on the International Tables of Glycaemic Index Values 2002, values were updated to current values using the International Tables of Glycaemic Index Values 2008 and other recent studies as previously described [19]. QBuilder software generates a frequency of consumption output as well as calculating average daily nutrient consumption and GI and GL values.

#### Statistical Analysis

Statistical analysis was carried out using Statistical Package for the Social Sciences (SPSS) software version 20.0 (SPSS Ltd., New York, NJ, USA). Statistical analyses involved: correlations for preliminary investigation of associations; chi-squared tests to examine relationships between categorical variables such as ethnicity and education level; independent sample *t*-tests to compare the control and intervention groups in relation to dietary and weight variables and also to compare the condensed and unmodified FFQs for validation; paired sample *t*-tests to compare the GI from the full FFQ taken in early pregnancy with the GI from the modified FFQ completed at 3 months post-partum; and ANOVA to examine the relationship between variables such as ethnicity and education level with 3 month dietary and weight variables. A *p*-value of <0.05 was considered statistically significant.

## 3. Results

Of the original 800 participants of the ROLO study, 749 completed the study and gave birth to eligible infants (healthy singleton births). 61.4% of these women (228 from the intervention group and 232 from the control group) returned the questionnaires at 3 months postpartum. Participants who returned questionnaires were significantly older (33.01 ± 3.91 *vs.* 31.96 ± 4.55 years, *p* = 0.001) and had a significantly higher pre-pregnancy weight (75.0 ± 14.9 *vs.* 72.6 ± 13.4 kg, *p* = 0.026) than those that did not; however, there was no difference in gestational weight gain (*p* = 0.290) or membership of the intervention group (*p* = 0.641) between them. There was no difference in demographic characteristics of the control and intervention groups (please see Table 1). Two hundred and twenty one offspring were boys and 239 girls (*p* = 0.944). There were 415 white Irish, 35 white non-Irish, 4 Chinese and 6 Filipino/South East Asian mothers who returned the questionnaires at 3 months postpartum and again, ethnicity was not significantly different between the intervention and control groups (*p* = 0.068). Education level was also similar between the intervention and control groups (*p* = 0.463). A comparison of control and intervention groups with regard to lifestyle and diet at 3 months postpartum is displayed in Table 2.

GI was significantly higher at 3 months post-partum than during the first half of pregnancy in both the intervention (54 ± 5 *vs.* 53 ± 4, *p* = 0.003) and control group (55 ± 5 *vs.* 53 ± 4, *p* < 0.001). The GL of the diet was significantly lower in the intervention group at 3 months postpartum than the control group (128 ± 49 *vs.* 145 ± 92, *p* = 0.014).

Women in the intervention group lost 1.3 ± 7.4 kg from early pregnancy to 3 months postpartum whereas women in the control group had gained weight (0.1 ± 5.2 kg). This weight change from early pregnancy to 3 months postpartum was statistically significant between the control and intervention groups (*p* = 0.022). However, there was no statistically significant difference in maternal weight between the control and intervention groups at 3 months postpartum (*p* = 0.708) nor was there a significant difference in weight loss from late pregnancy to 3 months postpartum between the groups (*p* = 0.651). When GI and GL at 3 months postpartum were examined in relation to maternal weight at 3 months postpartum and weight changes since pregnancy there was no association between 3 month GI and change in weight from baseline to 3 months postpartum (*p* = 0.809) but there was with 3 month GL and change in weight from baseline to 3 months postpartum (B = 0.010, *p* = 0.009). There was no association between either GI or GL and weight change between late pregnancy and 3 months postpartum (*p* = 0.930 and *p* = 0.750 respectively). Reported health behaviours of the intervention and control group at 3 months postpartum are displayed in Table 3. There was no difference between the control and intervention groups in reports of adherence to a weight-reducing diet at 3 months postpartum (*p* = 0.797), in breastfeeding commencement (*p* = 0.782) or duration (*p* = 0.876), or in reported levels of physical activity except for strenuous physical activity which was carried out on 0.44 ± 1.01 occasions per week by the intervention group and 0.67 ± 1.28 occasions per week by the control group (*p* = 0.044).

**Table 1:** Baseline characteristics of participants prior to dietary intervention and randomization to low GI or control diet in pregnancy.

Variable	Intervention *n* = 228	Control *n* = 232	Total *n* = 460	*p*-Value
Mother Age (years)	32.8 ± 4.0	33.2 ± 3.8	33.0 ± 3.9	0.275
Mother Weight at Booking (kg)	73.1 ± 13.9	72.2 ± 13.1	72.6 ± 13.5	0.514
Mother Height (cm)	165.5 ± 12.8	164.7 ± 16.8	165.1 ± 14.9	0.540
Mother BMI at Booking (kg/m^2^)	26.4 ± 4.7	26.2 ± 4.6	26.3 ± 4.7	0.636
Birthweight (kg)	4.1 ± 0.5	4.0 ± 0.5	4.0 ± 0.5	0.762
Mild Physical activity *	4.0 ± 2.6	4.1 ± 2.5	4.0 ± 2.5	0.728
Moderate Physical activity *	3.5 ± 2.5	3.0 ± 1.9	3.3 ± 2.2	0.081
Strenuous Physical activity *	2.2 ± 1.3	1.9 ± 0.7	2.0 ± 1.0	0.284
Pre-Randomisation Glycaemic Load	134 ± 34	139 ± 39	136 ± 36	0.170
Pre-Randomisation Glycaemic Index	58 ± 4	58 ± 4	58 ± 4	0.814

Independent sample *t*-tests were carried out and the results are expressed as mean ± standard deviation. *p*-value < 0.05 was considered statistically significant. * Number of 20 min intervals of physical activity per week at baseline—Strenuous physical activity was defined as physical activity that resulted in “rapid heartbeat”, moderate physical activity was defined as “not exhausting” and mild physical activity was defined as “minimal effort” physical activity. Relevant examples were provided for each.

**Table 2:** Comparison of intervention (low glycaemic index diet) and control groups at 3 months postpartum.

Variable	*N*	Intervention	*N*	Control	*t*-Value	*p*-Value
		Mean ± SD		Mean ± SD		
Glycaemic Load	228	128 ± 49	232	145 ± 92	−2.454	0.014
Glycaemic Index	228	55 ± 7	232	55 ± 7	−0.242	0.809
Mother weight 3 months (kg)	209	71.5 ± 14.6	208	72.0 ± 12.0	−0.374	0.708
Change in mother weight from baseline to 3 months (kg)	207	−1.3 ± 7.4	207	0.1 ± 5.2	−2.292	0.022
Mother weight in late pregnancy (kg)	95	87.6 ± 13.7	88	86.0 ± 14.4	0.795	0.428
Change in mother weight late pregnancy to 3 months (kg)	88	−14.3 ± 5.5	77	−13.9 ± 4.2	0.453	0.651
Days/week walking > 30 min	226	3.7 ± 2.3	229	3.7 ± 2.1	0.023	0.982
Mild PA 3 months *	208	3.6 ± 3.2	213	3.8 ± 3.0	−0.690	0.491
Moderate PA 3 months *	213	3.0 ± 3.0	220	3.0 ± 4.0	−0.119	0.905
Strenuous PA 3 months *	208	0.4 ± 1.0	217	0.7 ± 1.3	−2.017	0.044
Times/week attending gym	216	0.4 ± 1.0	221	0.4 ± 1.0	−0.235	0.814
Breastfeeding duration (weeks)	223	1.4 ± 1.3	227	1.5 ± 1.3	−0.156	0.876
Infant weight (kg)	211	7.0 ± 4.4	211	6.8 ± 1.0	0.759	0.448

Data was analysed using independent sample *t*-tests. A *p*-value of <0.05 was considered statistically significant. PA denotes physical activity. * Number of 20 min intervals of physical activity per week at baseline—Strenuous physical activity was defined as physical activity that resulted in “rapid heartbeat”, moderate physical activity was defined as “not exhausting” and mild physical activity was defined as “minimal effort” physical activity. Relevant examples were provided for each.

**Table 3:** Comparison of reported health behaviours of intervention (low glycaemic index diet) and control groups at 3 months postpartum.

Variable	*N*	Intervention	*N*	Control	*t*-Value	*p*-Value
		% with yes Response		% with yes Response		
Weight reducing diet 3 months	227	19.4	234	20.1%	0.258	0.797
Supplements 3 months	228	69.7	231	62.3	−1.675	0.095
Commenced breastfeeding	223	64.6	229	63.3	−0.277	0.782
Made dietary changes since ROLO study	207	69.1	213	61.5	−1.632	0.103
Low GI diet 3 months	104	23.1	93	4.3	−3.891	0.000
Reading food labels 3 months	224	82.1	229	73.8	−2.147	0.032
Reading ingredients	224	47.8	229	42.8	−1.062	0.289
Reading nutrients	224	64.3	229	53.3	−2.390	0.017
Reading calories	224	49.1	229	45.4	−0.786	0.432
Reading food weight	224	13.8	229	9.2	−1.559	0.120
Reading additives	224	32.6	229	24.5	−1.922	0.055
Reading serving size	224	13.4	229	9.6	−1.263	0.207
Attend gym	219	16.4	221	16.7	0.085	0.923

Data was analysed using independent sample *t*-tests and expressed as the frequency of participants that reported yes/no to the question of whether or not they participated in various health behaviours. A *p*-value of <0.05 was considered statistically significant. GI denotes glycaemic index.

The number of women who reported following a low GI diet at 3 months postpartum was significantly higher in the intervention group than the control group (24 *vs.* 4 women, *p* < 0.000) while more women reported reading food labels (184 *vs.* 169 women, *p* = 0.032). Of those who reported reading food labels, the only aspect of label-reading that differed between the groups was “reading nutrients” which was significantly higher in the intervention group (*p* = 0.017). 

## 4. Discussion

The intervention group had significantly higher weight loss from pre-pregnancy to 3 months postpartum than the control group. The intervention group reported higher levels of “following a low GI diet” and “reading food labels” and had a lower dietary glycaemic load (GL) than the controls.

The finding that the reduction in gestational weight gain observed in pregnancy [9] persisted at 3 months postpartum is likely to have an impact on maternal obesity in later years. A systematic review by Siega-Riz *et al.* [20] found that gestational weight gain in excess of the Institute of Medicine guidelines [7] resulted in higher postpartum weight retention both in the short, medium and long-term while a recent study by Knudsen *et al.* [15] found that high dietary GL in pregnancy was associated with increased postpartum weight retention at 18 months in overweight and obese women. Conversely, another recent study of low GI diet *vs.* traditional high-fibre dietary advice in GDM pregnancy found no difference in postpartum weight retention at 3 months. However, this study consisted of only 58 participants and extoled the need for adequately powered studies in this area [14]. While the difference in weight loss between the ROLO intervention and control groups from baseline to 3 months postpartum was modest and may not be of great clinical significance at present, it will be interesting to examine whether this difference persists postpartum over a greater time period. These findings also indicate the need for future studies of higher intensity interventions which may result in greater decreases in maternal postpartum weight. 

In the ROLO study participants, no significant difference was found in overall physical activity levels between the two groups at 3 months postpartum with the exception of strenuous physical activity which was at a higher level in the control group. It might be speculated that this was possibly in an attempt to lose the weight gained from early pregnancy to 3 months postpartum in comparison to the intervention group who had actually lost weight over this period. There is qualitative evidence from women in the postpartum period to illustrate that physical activity is deemed important for postpartum weight loss but perceive motherhood as a barrier to exercise [21]. However studies have found that pre-pregnancy lifestyle and physical activity levels are greater determinants of postpartum weight loss than motherhood transition [22].

Glycaemic load but not glycaemic index was significantly lower in the intervention group at 3 months postpartum and GL was also positively associated with change in maternal weight from baseline to 3 months postpartum. The lower GL, but not GI, could have resulted from avoidance of carbohydrate foods per se, possibly because high GI foods were being avoided, resulting in lower carbohydrate intake since calculation of GL is based on GI multiplied by actual carbohydrate content of foods consumed, as explained above [11]. There is very little research into adherence to low GI dietary advice post-intervention and all studies identified involved dietary guidance given for GDM. These studies found that in spite of knowledge of the risk of developing Type II diabetes, women failed to adhere postpartum to the dietary and lifestyle changes made during pregnancy [12,13,23]. In one of these studies by Stage *et al.* [13], although the women did not carry out recommended weight loss or major dietary change after pregnancy, those with a pre-pregnancy high fat diet did reduce their self-reported fat intake postpartum. 

When FFQs from early pregnancy and 3 months postpartum were compared, both the control and intervention groups had significantly higher GI diets postpartum than reported in early pregnancy. Authors were unable to find any other studies which had examined this and suggest that further research into this phenomenon is necessary to determine the cause.

Limitations of this study are that the modified FFQ was designed only to examine carbohydrate-containing foods and was validated against the full SLAN FFQ for glycaemic index alone. Therefore, diet in early pregnancy could only be compared with diet at 3 months postpartum for GI. However, since this was an intervention study, control and intervention groups could be compared at 3 months giving valuable data. A further limitation of this study was that postpartum weight and health behaviours were self-reported which may have resulted in bias. Although maternal weight was objectively measured during pregnancy and there was no difference in weight loss from measured late pregnancy weight to reported 3 months postpartum weight between the control and intervention groups, it is well-accepted that women, particularly those who are overweight or obese, tend to underreport weight reducing the reliability of this data [24]. Bias due to lack of an attention placebo for the control group may also have been an issue in this study as although the control group also received an the same number of ultrasound scans and completed the same anthropometric measurements and questionnaires as the control group, they did not receive the 1–2 h placebo session in trimester 1 nor any placebo information leaflets. Positive bias may also have been introduced through participation in the follow-up. However, this is the first study to report diet and lifestyle after a low GI intervention in pregnancy, particularly in a non-GDM cohort and therefore provides important information which has been absent from the scientific literature to date. In addition, increased reporting of food label reading demonstrates at least an improved awareness of health behaviours.

## 5. Conclusions

Low GI dietary intervention in pregnancy results in improved health-behaviours and continued reported compliance at 3 months postpartum possibly mediated through control of intake of carbohydrate-rich foods. Greater levels of weight loss from pre-pregnancy to 3 months postpartum in the intervention group may have important positive implications for overweight and obesity in later life.
